# Growth outcomes following recombinant human growth hormone therapy in Zhu–Tokita–Takenouchi–Kim syndrome

**DOI:** 10.1210/jcemcr/luag247

**Published:** 2026-09-16

**Authors:** Shien Chen Lee, Yusef Muflihi, Zainaba Mohammed, Esther Kinning, Renuka P Dias

**Affiliations:** Department of Paediatric Endocrinology and Diabetes, Birmingham Children's Hospital, Birmingham B46 NH, UK; Department of Paediatric Endocrinology and Diabetes, Birmingham Children's Hospital, Birmingham B46 NH, UK; Department of Paediatric Endocrinology and Diabetes, Birmingham Children's Hospital, Birmingham B46 NH, UK; West Midlands Regional Clinical Genetics Service, Birmingham Women's Hospital, Birmingham B15 2TG, UK; Department of Paediatric Endocrinology and Diabetes, Birmingham Children's Hospital, Birmingham B46 NH, UK; Department of Immunology and Immunotherapy, College of Medicine and Health, University of Birmingham, Birmingham B15 2TT, UK

**Keywords:** recombinant human growth hormone, Zhu–Tokita–Takenouchi–Kim syndrome, ZTTK, growth

## Abstract

Zhu–Tokita–Takenouchi–Kim (ZTTK) syndrome is a rare multisystem developmental disorder caused by heterozygous pathogenic variants in *SON*. Short stature is common, but data on endocrine function and response to recombinant human growth hormone (rhGH) are limited. We describe a female child with genetically confirmed ZTTK syndrome presenting with profound proportionate short stature, hypotonia, and developmental delay. Insulin-like growth factor 1 (IGF-1) of 91 ng/mL (Systeme international [SI]: 11.9 nmol/L; reference range, 33.6-170.5 ng/mL [SI: 4.4-22.3 nmol/L]) was normal, and peak growth hormone (GH) concentration on arginine stimulation testing was 7.2 ng/mL (SI: 21.6 mIU/L], above the UK diagnostic cutoff for GH deficiency (<6.7 ng/mL [SI: <20.1 mIU/L]). Given severely impaired linear growth and declining height velocity, a monitored trial of rhGH was initiated. Treatment with rhGH (0.035 mg/kg/day) resulted in >50% increase in height velocity from baseline (4.3-7.2 cm/year), suggesting a good response to rhGH, with sustained improvement over 2 years (height standard deviation score −4.16 to −3.40). Insulin-like growth factor 1, thyroid function, glucose, and glycated hemoglobin A1c remained normal. No adverse effects were observed. This case demonstrates a clinically significant growth response to rhGH in ZTTK syndrome despite absence of biochemical GH deficiency, supporting a supervised 12-month therapeutic trial.

## Introduction

Zhu–Tokita–Takenouchi–Kim syndrome (ZTTK syndrome, Online Mendelian Inheritance in Man [OMIM] #617140) is a rare, multisystem developmental disorder resulting from haploinsufficiency of the *SON* gene [1]. First described by Zhu et al in 2016 [2], ∼80 cases have been published, although the ZTTK SON-Shine Foundation estimates that more than 300 individuals worldwide may be affected [3]. Pathogenic variants arise predominantly *de novo*, with frameshift mutations accounting for most reported cases, followed by nonsense, missense, and deletion variants [4].

The *SON* gene plays a crucial role in RNA splicing and the regulation of gene expression [5]. *SON* haploinsufficiency disrupts multiple developmental pathways, resulting in the broad spectrum of phenotypes observed, including neurodevelopmental delay, hypotonia, brain malformations, visual impairment, skeletal anomalies, and feeding difficulties [4]. Although facial dysmorphism is frequently observed, no distinctive or consistent facial pattern is currently recognized for this condition [1].

Short stature is reported in about half of affected individuals, and growth hormone (GH) deficiency has been described in up to 14% [6]. Nevertheless, objective longitudinal data on growth trajectories are scarce, and evidence for recombinant human GH (rhGH) use remains limited [1, 7, 8].

We present a child with genetically confirmed ZTTK syndrome and profound short stature who demonstrated a marked improvement in linear growth following rhGH therapy, even without biochemical GH deficiency. This case provides valuable growth outcomes and highlights the potential utility of a trial of rhGH therapy in children with ZTTK syndrome who present with significant short stature.

## Case presentation

A 23-month-old girl was referred to pediatric endocrinology for severe faltering growth despite high-calorie nasogastric (NG) feeding. Her family history was unremarkable, with 2 healthy siblings of normal growth and development. Parents are from Black-African (Congolese) origin and nonconsanguineous.

Antenatal scans identified a muscular ventricular septal defect and asymmetrical ventriculomegaly, but the remainder of pregnancy was uncomplicated. She was born at 39 + 3 weeks via normal vaginal delivery with a birth weight of 2.65 kg (standard deviation score [SDS] −1.55), appropriate for gestational age by weight. Birth length and head circumference were not available from clinical records (and not routinely measured in the United Kingdom at birth).

Following delivery she had a 3-week neonatal admission for pulmonary hypertension and respiratory distress, requiring continuous positive airway pressure and feeding support via NG feeds. Hypotonia was also noted. Initial investigations were normal including electromyography. She was discharged home with NG feeding.

At 3 months of age, she developed severe bronchiolitis requiring intubation and mechanical ventilation in the pediatric intensive care unit. Over the next 2 years, she had 8 further respiratory infections requiring hospital admissions without intensive care. She was noted to have developmental delay by the age of 6 months. Her respiratory issues gradually improved with age. She had spontaneous closure of her ventricular septal defect.

## Diagnostic assessment

**Figure 1:**
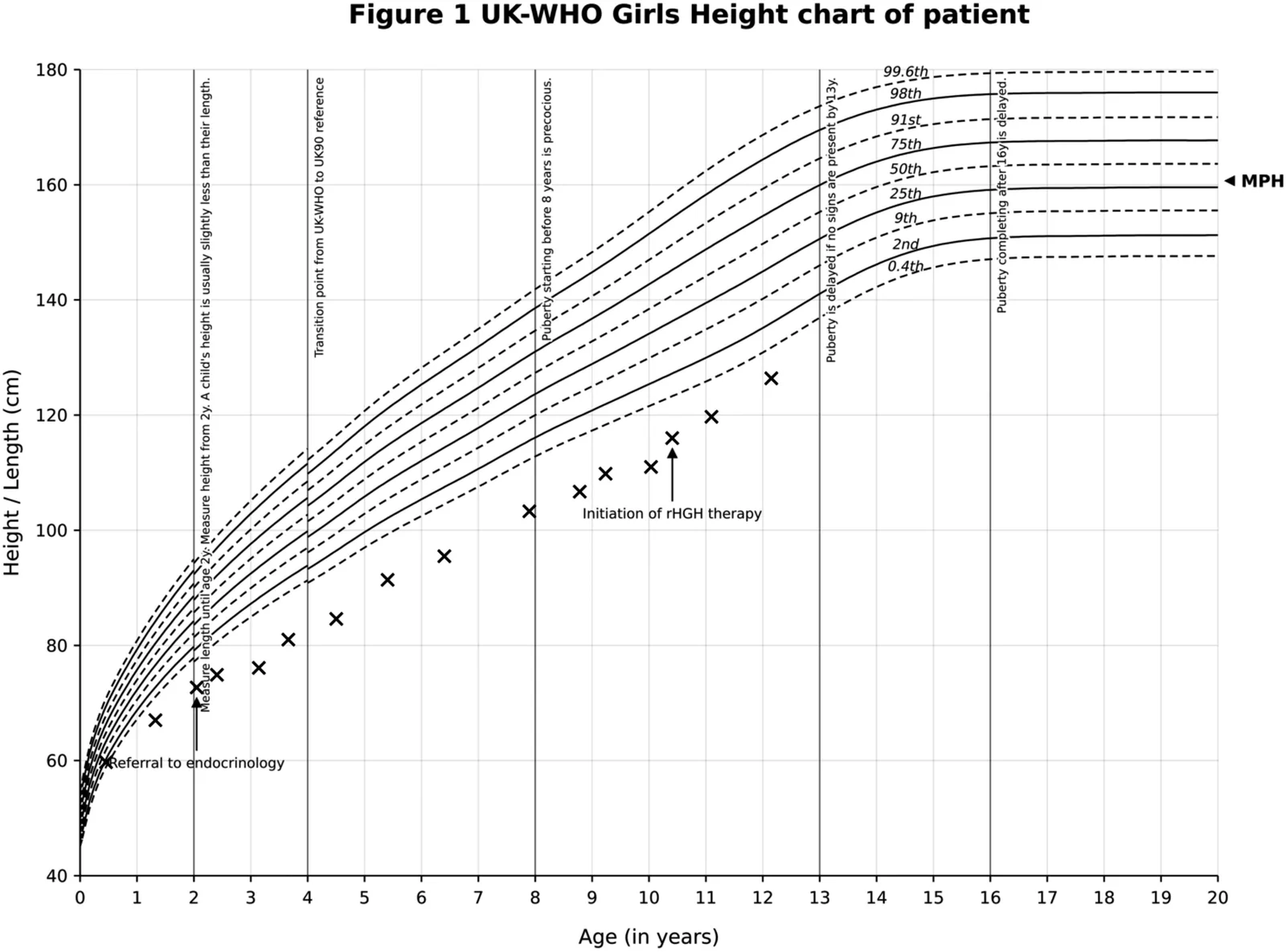
Height chart of patient. Growth chart derived from Electronic Health Record (based on UK-WHO growth charts). Boxes indicated key events (referral and initiation of rhGH) as well as MPH. Crosses indicate growth measurements taken in clinic.

During initial endocrine assessment at 23 months, height was 72.7 cm (SDS −4.0) and weight 7.3 kg (SDS −4.0), markedly below her mid-parental height (MPH; Fig. 1). She was receiving dietetic support with overnight 3-hourly NG feeds of Infatrini and Duocal (up to 160 mL/kg/day), in addition to 3 small solid meals per day.

Craniofacial features consisted of frontal bossing, a triangular face, low-set ears, a flat nasal bridge, and prominent eyebrows with long curled eyelashes. Limb anomalies included a short fifth finger, absent toenails on the fourth and fifth toes, and poor nail growth. No skin lesions or spinal abnormalities were present.

Developmental delay was evident from early infancy, with hypotonia, delayed motor milestones (head support by 8 months of age, sat without support by 11 months, independent walking after age 3), and absent expressive language.

Investigations at 23 months of age were unremarkable, with a full blood count, renal and liver function, bone profile and inflammatory markers all within normal limits. She has negative celiac serology and normal thyroid function tests, with free thyroxine 1.35 ng/dL (Systeme international [SI]: 17.4 pmol/L; reference range, 0.8-1.8 ng/dL; SI: 10.8-22.9 pmol/L) and thyroid-stimulating hormone 1.14 mIU/L (reference range, 0.5-3.8 mIU/L). Repeat bloods at 3.6 years showed a normal insulin-like growth factor 1 (IGF-1) of 91 ng/mL (SI: 11.9 nmol/L; reference range, 33.6-170.5 ng/mL [SI: 4.4-22.3 nmol/L]) and thyroid function test results remained normal (free thyroxine 1.31 ng/dL [SI: 16.9 pmol/L], thyroid-stimulating hormone 1.49 mIU/L).

As height remained persistently below her MPH, GH stimulation testing with arginine was performed at 5.4 years. Peak GH was 7.2 ng/mL (SI: 21.6 mIU/L), above the diagnostic cutoff for GH deficiency in the United Kingdom (<6.7 ng/mL [SI: <20.1 mIU/L]) [9], therefore ruling out this diagnosis. Adrenal reserve was adequate on a short synacthen test, with a cortisol peak of 57.9 µg/dL [(SI: 1600 nmol/L; local reference range for adequate response >18 µg/dL [SI: >500 nmol/L]).

Magnetic resonance imaging of the brain showed gray matter heterotopia with normal midline structures. Skeletal survey showed no evidence of skeletal dysplasia. Bone age assessment was not performed in this case. Genetic investigations, including microarray, developmental disorder panels and intellectual disability panels were unremarkable. She was reviewed in endocrine clinic every 12 to 18 months, with closer follow-up at 7.9 years after she developed premature adrenarche, to assess for progression to precocious puberty. Whole genome sequencing subsequently identified a *de novo* pathogenic heterozygous variant in *SON* (NM_138927.2) c. 2107del, p (Val703*), confirming a diagnosis of ZTTK syndrome at the age of 10 years.

## Treatment

Given her severe short stature and poor height velocity, the endocrine multidisciplinary team agreed that a 12-month trial of rhGH was appropriate to consider, to try and improve her linear growth.

At age 10 years, rhGH (somatropin) was commenced at 0.6 mg daily (0.035 mg/kg/day) and later increased to 0.7 mg to maintain equivalent dosing. This dose was selected as a conservative starting point within the National Institute for Health and Care Excellence (NICE) recommended dosing spectrum, reflecting the patient's baseline IGF-1 at the higher end of the normal range and the uncertainty of GH response in this rare genetic condition. Dose titration was planned based on clinical response and IGF-1 monitoring. Treatment was well tolerated with no adverse effects.

## Outcome and follow-up

Over 2 years of rhGH therapy, the patient demonstrated a marked improvement in linear growth (Fig. 1), with height SDS improving from −4.16 to −3.4 and modest gains in weight (Table 1). Prior to commencing rhGH therapy at age 9.7 years, height velocity was 4.3 cm/year. Following initiation of treatment, height velocity increased to 7.2 cm/year at age 10.4 years, an absolute improvement of 2.9 cm/year, representing a 67% increase from baseline. This indicated a good response to rhGH treatment [10]. At her most recent review (aged 12 years), she remained Tanner stage 1 with no breast development (B1, breast stage 1), suggesting that her improved height velocity was not attributable to a pubertal growth spurt. Pubic hair stage 3 (P3) and axillary hair stage 2 (A2) were consistent with progressive adrenarche.

**Table 1:** Summary of growth and pubertal assessment with IGF-1 levels

Age	IGF-1	Pubertal status*^a^*	Height velocity	Height	Height SDS	Weight	Weight SDS
7.89 years	Not available	B1, A1, P2	5.3 cm/year	1.033 m	−4.31 SD	14.9 kg	−4.13 SD
8.70 years	139.2 ng/mL (18.2 nmol/L)	B1, A1, P2	4.2 cm/year	1.067 m	−4.29 SD	15.5 kg	−4.44 SD
9.71 years	168.3 ng/mL (22.0 nmol/L)	B1, A1, P2+	4.3 cm/year	1.110 m	−4.16 SD	18.6 kg	−3.58 SD
10.40 years*^b^*	273.0 ng/mL (35.7 nmol/L)	B1, A1, P2+	7.2 cm/year	1.160 m	−3.76 SD	19.5 kg	−3.60 SD
11.09 years*^b^*	189.0 ng/mL (24.7 nmol/L)	B1, A1, P2–3	5.4 cm/year	1.197 m	−3.60 SD	20.8 kg	−3.50 SD
12.15 years*^b^*	378.6 ng/mL (49.5 nmol/L)	B1, A2, P3	6.4 cm/year	1.264 m	−3.40 SD	24.7 kg	−2.99 SD

Insulin-like growth factor 1 reference ranges based on Tanner staging: Stage 1: 77.2-235.6 ng/mL (SI: 10.1-30.8 nmol/L); Stage 2: 120.8-449 ng/mL (SI: 15.8-58.7 nmol/L); Stage 3: 180.5-522.4 ng/mL (SI: 23.6-68.3 nmol/L).

Abbreviations: IGF-1, Insulin-like Growth Factor 1; SDS, standard deviation score.

^*a*^Based on Tanner staging: B, breast stage; A, axillary hair stage; P, pubic hair stage.

^*b*^After initiation of rhGH therapy.

Biochemical monitoring demonstrated stable IGF-1 within the reference range (Table 1), normal thyroid function and normal fasting glucose and hemoglobin A1c. Her mother and school staff reported improvements in stamina, physical appearance, and functional participation.

## Discussion

Short stature is a recognized feature of ZTTK syndrome, although reported prevalence varies across studies. A 2021 cohort found short stature in 54% of individuals [1], while a more recent literature review reported a lower prevalence of 29.11% [11]. Growth impairment may begin prenatally. Intrauterine growth restriction was documented in 5 of 7 individuals in one cohort [12], and the first prenatal diagnosis of ZTTK syndrome was made following trio whole-exome sequencing for early-onset fetal growth restriction associated with corpus callosum dysgenesis, cortical abnormalities, and gastrointestinal malformations [13].

Postnatally, feeding difficulties are common and can exacerbate growth faltering, with some children requiring gastrostomy tube support [12, 14]. Growth may also be influenced by underlying neurological and structural abnormalities. Brain malformations are common in ZTTK syndrome, with neuroimaging findings including ventriculomegaly, a thin corpus callosum, cortical or cerebellar atrophy, and cortical anomalies [1]. Notably, in one study evaluating brain magnetic resonance imaging in 7 affected individuals, 5 demonstrated a small pituitary gland with a markedly elongated pituitary stalk [15], suggesting possible hypothalamic–pituitary involvement in their poor growth.

Growth hormone deficiency has been described in ZTTK syndrome, with an estimated prevalence of up to approximately 14% [6]; however, the true frequency remains unknown due to limited endocrinologic data. Although GH deficiency may contribute to growth impairment in some individuals, this is not universally observed, suggesting that the variable growth outcomes seen in ZTTK syndrome are multifactorial.

Evidence on the efficacy of rhGH therapy in ZTTK syndrome remains extremely limited. Two published case reports describe individuals with ZTTK syndrome and GH deficiency, although neither provides details on treatment or longitudinal growth outcomes [14, 16]. Notably, both reports defined GH deficiency as a peak GH response of <15 ng/mL (SI: 45 mIU/L) on stimulation testing, which is substantially higher than the current UK diagnostic cutoff for GH deficiency diagnosis and rhGH therapy eligibility of <6.7 ng/mL (SI: <20.1 mIU/L). Such discrepancies in diagnostic thresholds across studies likely reflect differences in GH immunoassays and stimulation protocols used, further complicating cross-study comparison [9]. An additional case described a 15-year-old girl with ZTTK syndrome who received rhGH for short stature, however pre- and post-treatment height data were not available, limiting interpretation of therapeutic benefit [7].

A retrospective review identified 3 affected children who reportedly showed improvement with rhGH treatment [1]; however, specific growth outcomes and GH stimulation results were not available. More recently, an abstract described a 5-year-old with ZTTK syndrome and GH deficiency who demonstrated minimal response to rhGH despite 5 years of relatively high-dose treatment (0.05 mg/kg/day), with height SDS changing only from −4.05 to −3.98 [8].

In contrast, our patient demonstrated a substantial and sustained improvement in height velocity and SDS despite not meeting UK diagnostic criteria for GH deficiency. To our knowledge, this is the first reported case of an individual with ZTTK syndrome, a normal GH response on stimulation testing, and a clear linear growth response to rhGH therapy. This finding suggests that rhGH may offer therapeutic benefit for children with ZTTK syndrome who have significant short stature even in the absence of GH deficiency. Based on this experience, we propose that a monitored 12-month trial of rhGH therapy could be considered within the existing surveillance framework for children with ZTTK syndrome and severe short stature and poor growth [1], supervised by an appropriate pediatric team.

This case has several limitations. Bone age was not assessed, as the clinical phenotype, including dysmorphic features, global developmental delay, and severe short stature markedly below her MPH was consistent with an underlying genetic syndrome rather than constitutional delay of growth and puberty. As a single case report, our findings cannot be generalized, and the growth response observed may not be representative of all individuals with ZTTK syndrome. Furthermore, the absence of standardized endocrine evaluation across published ZTTK cohorts, combined with inter-assay variability in GH measurement, limits direct comparison with other reported cases.

Given the scarcity of published growth data, this case contributes meaningful clinical insight and underscores the need for more systematic evaluation of endocrine involvement in individuals with ZTTK syndrome.

## Learning points

- Short stature is relatively common in ZTTK syndrome, and the growth impairment seen may be multifactorial, arising from prenatal factors, feeding difficulties, neurodevelopmental impairment, and potential hypothalamic–pituitary involvement.
- Response to rhGH appears variable, reflecting underlying genetic and metabolic heterogeneity.
- A carefully monitored trial of rhGH may be a reasonable consideration in selected patients with severe, proportionate short stature, even in the absence of classical GH deficiency.
- Long-term follow-up is essential to evaluate efficacy, safety, and the impact on functional outcomes.

## Contributors

All authors made individual contributions to authorship. R.P.D. and E.K. were involved in the clinical diagnosis and management of this patient. Z.M., S.C.L., and Y.M. contributed to clinical data acquisition. R.P.D., S.C.L., and Y.M. collected and analyzed the results and wrote the case report. Z.M. contributed to manuscript revision. All coauthors gave feedback and approved the final version of the report. The family also reviewed and approved the submitted manuscript.

## Data Availability

Original data generated and analyzed during this study are included in this published article.

## Abbreviations

GH: growth hormone
IGF-1: insulin-like growth factor 1
MPH: mid-parental height
NG: nasogastric
rhGH: recombinant human growth hormone
SDS: standard deviation score
ZTTK: Zhu–Tokita–Takenouchi–Kim
